# Epidemiology of Infections with SARS-CoV-2 Omicron BA.2 Variant, Hong Kong, January–March 2022

**DOI:** 10.3201/eid2809.220613

**Published:** 2022-09

**Authors:** Yonatan M. Mefsin, Dongxuan Chen, Helen S. Bond, Yun Lin, Justin K. Cheung, Jessica Y. Wong, Sheikh Taslim Ali, Eric H.Y. Lau, Peng Wu, Gabriel M. Leung, Benjamin J. Cowling

**Affiliations:** World Health Organization Collaborating Centre for Infectious Disease Epidemiology and Control, School of Public Health, Li Ka Shing Faculty of Medicine, The University of Hong Kong, Hong Kong, China (Y.M. Mefsin, D. Chen, H.S. Bond, Y. Lin, J.K. Cheung, J.Y. Wong, S.T. Ali, E.H.Y. Lau, P. Wu, G.M. Leung, B.J. Cowling);; Laboratory of Data Discovery for Health, Hong Kong Science and Technology Park, Hong Kong (D. Chen, S.T. Ali, E.H.Y. Lau, P. Wu, G.M. Leung, B.J. Cowling)

**Keywords:** COVID-19, coronavirus disease, SARS-CoV-2, severe acute respiratory syndrome coronavirus 2, viruses, respiratory infections, zoonoses, vaccine-preventable diseases, Hong Kong, China, epidemiology, fatality, Omicron, BA.2

## Abstract

Our analysis of data collected from multiple epidemics in Hong Kong indicated a shorter serial interval and generation time of infections with the SARS-CoV-2 Omicron variant. The age-specific case-fatality risk for Omicron BA.2.2 case-patients without complete primary vaccination was comparable to that of persons infected with ancestral strains in earlier waves.

Several SARS-CoV-2 variants of concern have caused large outbreaks of infection, including deaths, after the emergence of the ancestral strain in late 2019. First detected in South Africa in November 2021, the Omicron variants quickly became dominant across the world, even in countries with high SARS-CoV-2 vaccination coverage (*1*), probably attributable to enhanced immune escape and increased transmissibility (*2*).

Hong Kong (population 7.4 million), a special administrative region of China, applied intensive public health and social measures to control 4 epidemic waves during 2020–2021, in which 9,403 locally infected (nonimported) cases (1.3 cases/1,000 population) and 207 fatalities occurred. During December 31, 2021–May 21, 2022, a total of 9,148 deaths and >1 million cases largely caused by Omicron were reported in the fifth pandemic wave (Figure 1, panel A, B). The COVID-19 vaccination program in Hong Kong began in late February 2021 and uses the mRNA vaccine BNT162b2 (Pfizer-BioNTech, https://www.pfizer.com) and the inactivated vaccine CoronaVac (Sinovac, https://www.sinovac.com). Approximately 10% of the population had been vaccinated with 1 dose by late April 2021, and coverage slowly increased thereafter. The Omicron variant (BA.1) was first detected in Hong Kong among 2 travelers in hotel quarantine in November 2021 (*3*), and a small community outbreak occurred in early January 2022, linked to 2 aircrew members infected overseas (*4*). Subsequently, Omicron BA.2.2 cases were reported in another quarantine hotel in mid-January in an arriving traveler who was reaching the end of a 21-day quarantine (*5*). Ultimately, a large fifth wave dominated by BA.2.2 peaked in early March 2022 after rising exponentially for >1 month, with a doubling time of 3.1 days (Figure 1, panel C). Virus sequencing conducted throughout the epidemic indicated that the last local BA.1 cases were detected in mid-January and 1 sporadic local Delta detection occurred in late March (Leo Poon, University of Hong Kong, pers. comm., email, May 28, 2022).

**Figure 1:**
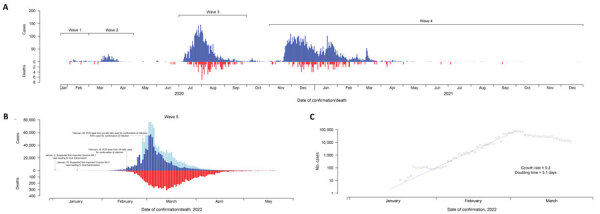
Confirmed COVID-19 local cases and deaths in Hong Kong over the first 4 epidemic waves (A) and wave 5 (B, C). Blue and red bars in panels A and B indicated daily numbers of COVID-19 cases confirmed by reverse transcription PCR and daily number of reported deaths. Bars in light blue in panel B indicate daily numbers of COVID-19 cases detected by rapid antigen tests. Panel C indicates the exponential growth of the reported COVID-19 cases in wave 5 before the epidemic peaked. Six fatal imported cases were also included in panel A; no deaths were reported among persons with imported cases in wave 5.

## The Study

We analyzed contact-tracing data on reverse transcription PCR–confirmed COVID-19 cases reported during December 31, 2021–January 22, 2022, to estimate the serial interval and generation time for Omicron (Appendix). Given the 207 deaths that occurred in 9,403 locally infected case-patients in the first 4 epidemic waves and 106 deaths among 4,604 case-patients discharged from hospital isolation in the early period of wave 5, we estimated the age-specific case-fatality risk (CFR) for case-patients infected with ancestral strains compared with Omicron-infected case-patients.

By using information on 80 case-patients (57 infected with BA.1 and 23 with BA.2) with known exposure and symptom onset information, we estimated the mean (+SD) incubation periods to be 4.58 (+1.72) days for Omicron BA.1 and 4.42 (+1.42) days for Omicron BA.2 (Appendix Table 1). For 43 symptomatic infector–infectee pairs, we estimated the mean (+SD) serial interval for BA.1 infections (n = 30) as 3.30 (+1.95) days; median was 3.17 days. For BA.2 (n = 13), the estimated mean (+SD) serial interval was 2.72 (+1.51) days; median was 2.52 days. We used gamma distribution for estimates of BA.2 accounting for the potential for epidemic phase bias (*6*) with a growth rate of 0.25, because data were collected during the early growth phase of the BA.2 epidemic.

**Figure 2:**
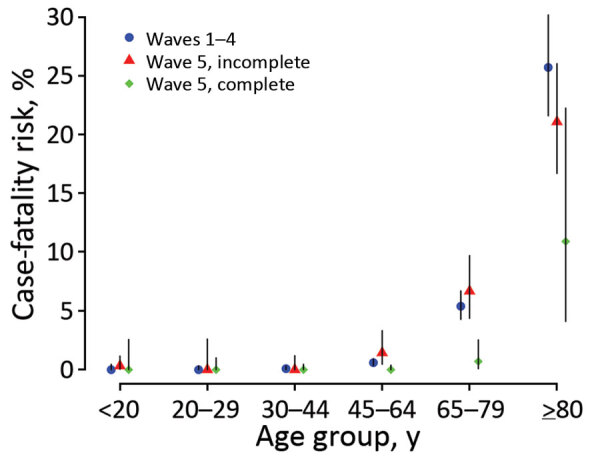
Age-stratified estimates of the case-fatality risk for COVID-19 in epidemic waves 1–4 and wave 5 in Hong Kong by vaccination status. Case-patients were classified as having a complete primary series if they had received >2 doses of COVID-19 vaccines >2 weeks before symptom onset (for symptomatic case-patients) or >3 weeks before laboratory confirmation of the infection (for symptomatic case-patients with a missing onset date or asymptomatic case-patients), otherwise as having an incomplete primary series. The COVID-19 vaccines available in Hong Kong included BNT162b2 (Pfizer-BioNTech, https://www.pfizer.com) and CoronaVac (Sinovac, https://www.sinovac.com) vaccines.

In the early period of the fifth wave (on or before February 15, 2022), all reported COVID-19 cases were only confirmed through reverse transcription PCR conducted by Hong Kong’s Public Health Laboratory Services, as in earlier waves (Figure 1; Appendix Figure 1). After accounting for unresolved outcomes in some persons, we estimated that the age-specific CFR for case-patients without completion of a primary series of vaccination in wave 5 was comparable to that of case-patients confirmed in waves 1–4 across all age groups (Figure 2; Appendix Table 2). The highest fatality risk observed in waves 1–4 for patients >80 years of age (24.9% [95% CI 20.9%–29.3%]) was similar to that for unvaccinated persons at the same age from wave 5 (21.7% [95% CI 17.1%–26.8%]) (Figure 2). The CFR for persons >80 years of age who had not completed a primary series of vaccination in wave 5 was approximately double the risk for persons in the same age group who had completed a primary series (11.1% [95% CI 4.2–22.6]). Among case-patients 65–79 years of age, the CFR was 5.2% (95% CI 4.1%–6.5%) in waves 1–4, 6.7% (4.3%–9.8%) in wave 5 with incomplete primary series, and 0.7% (0.1%–2.4) with complete primary series (Figure 2; Appendix Table 2). The 8 deaths that occurred in adults >65 years of age with a complete primary series of vaccination were all in persons who had received 2 or 3 doses of CoronaVac vaccine.

## Conclusions

Our study estimated a relatively shorter serial interval and generation time of the Omicron BA.2 subvariant in Hong Kong compared with earlier variants (*7*), which would have contributed to faster spread in the population along with the higher intrinsic transmissibility. Our analysis was conducted on a relatively small number of case pairs in the fifth wave. Similar studies conducted in South Korea and the Netherlands reported shorter mean incubation periods as low as 3.2 days (*8*) and serial intervals of 2.8–3.0 days (*9*,*10*). The peak of the fifth epidemic in Hong Kong in early March despite no major change in social distancing measures probably indicates sufficient infections to create herd immunity, at least temporarily, with subsequent infections overshooting that threshold (*11*).

Among all the deaths in persons whose age was recorded through May 21 in Hong Kong, 92.7% (8,482/9,146) occurred in persons >65 years of age and 71.1% (6,500/9,146) in persons >80 years of age. We found a generally similar fatality risk for unvaccinated case-patients across age groups in the early period of the fifth wave compared with earlier waves, although the CFR for Omicron cases in person >80 years of age without complete primary vaccination series might be slightly lower than persons infected with ancestral strains. This finding indicates that the intrinsic severity of BA.2 may not be much lower than the ancestral strain. Nonetheless, only 106 fatal cases that occurred in the fifth wave were applied in the analysis. Infections with the Omicron variant were reported to have milder severity in South Africa (*12*) and elsewhere (*13*,*14*), where most of the population were either exposed to previous infections or had been vaccinated. However, our estimates might slightly overestimate the fatality risk of Omicron in Hong Kong because a small number of cases of Delta infection, including fatal cases, might have been included in the analysis and some milder COVID-19 cases might not have been diagnosed and isolated yet during the early period of the fifth wave. 

The relatively high number of deaths in Hong Kong’s fifth wave can be attributed to the high incidence of infections within a short period and the low level of vaccination coverage in older adults. Although the overall vaccination coverage was 70% at the start of the fifth wave, only 50% of persons >65 years of age and 20% of persons >80 years of age had completed a primary series of vaccination. Vaccine hesitancy in older adults in Hong Kong appeared to be associated with low confidence in the government and the concern about the risk for adverse events after vaccination among persons with underlying medical conditions (*15*). Overall, our findings highlight the importance of achieving high vaccination coverage, especially in older adults, and the need to reassess public health and social measures in response to any more transmissible SARS-CoV-2 variant in the future.

Appendix Additional information about epidemiology of infections with SARS-CoV-2 Omicron BA.2 variant, Hong Kong, January–March 2022.
