# Supplementary material for: Epidemiology of Infections with SARS-CoV-2 Omicron BA.2 Variant, Hong Kong, January–March 2022
Source: Emerg Infect Dis. 2022 Sep;28(9):1856–8. doi: 10.3201/eid2809.220613 (PMC9423929; doi:10.3201/eid2809.220613)
Supplement: Appendix — Additional information about epidemiology of infections with SARS-CoV-2 Omicron BA.2 variant, Hong Kong, January–March 2022. [file 22-0613-Techapp-s1.pdf]

Article DOI: https://doi.org/10.3201/eid2809.220613

Epidemiology of Infections with SARS-CoV-2 Omicron BA.2 Variant, Hong Kong, January–March 2022

Appendix

Data Sources

We obtained all confirmed cases with SARS-CoV-2 infections in Hong Kong from the Department of Health and the Hospital Authority of the Government of Hong Kong Special Administrative Region from 23 January 2020 to 17 March 2022. Case information includes age, sex, time of symptom onset, sample collection, laboratory confirmation, report, admission, discharge and death, severity status (mild-to-moderate, severe or critical, fatal), SpO_2 levels, time and vaccination and type of vaccine for each dose. We also collected contact tracing data published by the Government or from media reports that quoted government statements on some of the confirmed case clusters (*1*). The contact tracing data provided information on demographics (age and sex), date of exposure, symptom onset date, case category (symptomatic /asymptomatic), type of SARS-CoV-2 variant, and vaccination status. The confirmed cases in a cluster either had a single exposure date or a reported interval of exposure defined by the earliest and latest dates of exposure to an index case. In Hong Kong a COVID-19 case can be confirmed by the real-time reverse transcription-polymerase chain reaction (real-time RT-PCR) assay (since the emergence of SARS-CoV-2 in 2020) or by a rapid antigen test (RAT) since 26 February 2022.

Epidemiologic Parameters Definition

We define that the incubation period is the time interval between initial contact with a confirmed SARS-CoV-2 case and symptom onset, serial interval is the time interval between symptom onset of the infector and the infectee in a transmission pair, and generation time is the time interval between exposure time of the infector and the infectee in a transmission pair.

Transmission Pair Construction

We constructed infector-infectee transmission pairs with laboratory-confirmed SARS-CoV-2 omicron infection using contact tracing data published on the Web site of the Department of Health, the Government of the Hong Kong Special Administrative Region, and media reports that quoted government statements.

We constructed transmission pairs for estimating mean serial interval and generation time if the infector had direct contact with its infectee. A transmission pair was defined as two confirmed COVID-19 cases identified in the epidemiologic investigation by showing a clear epidemiologic link with each other, which is the infector needs to have direct contact with the infectee. The 'infector' was defined as the primary case, with an identified source of exposure occurring before their encounter with the 'infectee'. The 'infectee' was defined as the secondary case whose exposure was solely by the infector. In the same chain of transmission, the same infector could generate more than one pair if they infected more than one individual. In a large case cluster with multiple generations of transmission, an infectee in a transmission pair might also be an infector in another pair.

For serial interval calculation, as there is established evidence of asymptomatic/ presymptomatic transmission of SARS-CoV-2 (*2,3*), infector-infectee pairs were determined regardless of the order of their onset dates. The infectors-infectees order were determined by who were first exposed to suspected source of infection and induced further infection spread to his/her own network, especially in the case of household or workplace transmission setting. However, to be conservative, in a cluster where the serial interval between index case to off-spring case was larger than 6 days (C. Kremer et al., unpub. data, https://doi.org/10.1101/2022.01.28.22269756), such transmission pairs were excluded as this was suggestive of possible intermediate transmission.

For exposure time that was not explicitly recorded in the government document, we constructed exposure interval based on the following assumptions:

1. The latest exposure time (lower bounds of the exposure interval) is symptom onset date or isolation/quarantine date or confirmation date (if asymptomatic) of the infector

2. The earliest exposure time (upper bounds of the exposure interval) is the infector's exposure date if the infector was a local family member or the infector's arrival date if the infector was an imported family member.

Statistical Analysis

Estimation of Serial Interval and Generation Time

We fitted the parametric distributions of Lognormal, Weibull, and Gamma models to the time intervals data and estimated the distributions of incubation period, serial interval and generation time using the maximum likelihood method (*4,5*) (N. Gozzi et al., unpub. data, https://doi.org/10.1101/2022.01.04.22268721). We accounted for the interval censoring of exposure windows in estimation of the incubation period and generation time. The best fitted model was determined by the smallest value of Akaike's Information Criterion. Estimates of the mean, standard deviation (SD), median and 95% percentiles were derived from the models. The corresponding 95% confidence intervals (CIs) of each estimate were constructed using the parametric bootstrap method with 1000 bootstrapped samples. Because data on serial interval included zero value, we shifted data by adding 1 day to each serial interval so that we could fit distributions. Furthermore, Omicron BA.2 parameters estimations were also made using gamma distribution accounting for the sampling bias that could happen when parameters estimation made using data collected during the initial growth phase of the outbreak (*6*). In our calculation, sampling bias correction was made based on the computed exponential growth rate ($r = 0.25$) of BA.2 using data collected from 15–23 January 2022.

Estimation of Case-Fatality-Risk (CFR)

The COVID-19 case line list obtained from the Hospital Authority was used for estimation of the CFR. Cases confirmed after 15/02/2022 in the fifth wave were excluded from the analysis, and fatal cases were identified with the date of death indicated in the dataset as of 23 March 2022. COVID-19 patients who were not fatal and with a date of discharge were defined as recovered cases. Hong Kong has started COVID-19 vaccination programme since later February 2021 which allow us to analyze the CFR in patients with and without vaccination. Cases who had two or more doses of the mRNA vaccine BNT162b2 (BioNTech/Fosun Pharma/Pfizer) and the inactivated vaccine CoronaVac (Sinovac) ≥14 days before symptom

onset were classified as completing the primary vaccination series while the rest including without vaccination or being vaccinated with 1 dose or 2 doses within 14 days before onset were referred to as incomplete primary vaccination series. Cases without information on the vaccination status or age were excluded from the analysis.

In estimation of the CFR, we classified all the COVID-19 cases confirmed on or before 15 February into three groups, i.e., cases occurred in Waves 1 to 4 (before 01/01/2022), and cases with a complete and incomplete primary vaccination series for the early period of Wave 5 (between 01/01/2022 and 15/02/2022). The CFR was then calculated for six age groups (<20, 20–29, 30–44, 45–64, 65–79, 80+ years) considering vaccination status of cases with the equation: CFR = death/ (death + recovered) allowing for the potential delay from admission to death. The confidence intervals were calculated using a binomial (n,p) test with n = the number of deaths and p = the number of deaths + the number of recovered cases.

References

1. Center for Health Protection Department of Health. Press releases: The Government of the Hong Kong Special Administrative Region. 2022 [cited 2022 March 29]. https://www.chp.gov.hk/en/media/116/index.html

2. Qian G, Yang N, Ma AHY, Wang L, Li G, Chen X, et al. COVID-19 transmission within a family cluster by presymptomatic carriers in China. Clin Infect Dis. 2020;71:861–2. PubMed https://doi.org/10.1093/cid/ciaa316

3. Johansson MA, Quandelacy TM, Kada S, Prasad PV, Steele M, Brooks JT, et al. SARS-CoV-2 transmission from people without COVID-19 symptoms. JAMA Netw Open. 2021;4:e2035057. PubMed https://doi.org/10.1001/jamanetworkopen.2020.35057

4. Cauchemez S, Fraser C, Van Kerkhove MD, Donnelly CA, Riley S, Rambaut A, et al. Middle East respiratory syndrome coronavirus: quantification of the extent of the epidemic, surveillance biases, and transmissibility. Lancet Infect Dis. 2014;14:50–6. PubMed https://doi.org/10.1016/S1473-3099 (13)70304-9

5. Cowling BJ, Park M, Fang VJ, Wu P, Leung GM, Wu JT. Preliminary epidemiological assessment of MERS-CoV outbreak in South Korea, May to June 2015. Euro Surveill. 2015;20:7–13. PubMed https://doi.org/10.2807/1560-7917.ES2015.20.25.21163

6. Britton T, Scalia Tomba G. Estimation in emerging epidemics: biases and remedies. J R Soc Interface. 2019;16:20180670. PubMed https://doi.org/10.1098/rsif.2018.0670

Appendix Table 1. Estimated mean and percentiles of incubation period, serial interval and generation time of infections with SARS-CoV-2 Omicron BA.1 and BA.2 subvariants, 31 December 2021 to 22 January 2022, Hong Kong.

Omicron subvariant	Parameters (sample size)	Best fitted distribution	Mean (95% CI)	SD (95% CI)	Median (95% CI)	95 percentile (95% CI)
BA.1	Incubation period (57)	Gamma	4.58 (4.1–5.08)	1.72 (1.32–2.07)	4.38 (3.88–4.87)	7.73 (6.72–8.71)
	Serial Interval (30)	Weibull	3.30 (2.65–4.01)	1.96 (1.42–2.41)	3.15 (2.49–3.92)	6.76 (5.36–8.16)
	Generation time (45)	Weibull	2.36 (2.01–2.77)	0.59 (0.38–0.90)	2.38 (2.01–2.80)	3.28 (2.79–3.97)
BA.2	Incubation period (23)	Weibull	4.03 (3.19–4.80)	1.12 (0.46–1.51)	4.05 (3.21–4.84)	5.82 (4.37–6.76)
		Gamma*	4.42 (3.40–5.21)	1.42 (0.36–1.99)	4.27 (3.29–5.02)	6.93 (4.85–8.80)
	Serial Interval (13)	Weibull	2.23 (1.53–2.90)	1.26 (0.72–1.63)	2.17 (1.46–2.91)	4.40 (2.99–5.31)
		Gamma*	2.72 (1.80–3.88)	1.51 (0.76–2.43)	2.52 (1.68–3.55)	5.50 (3.31–8.25)

* Estimation accounted for the potential epidemic phase bias since Omicron BA.2 were data collected during the early growth phase of BA.2 outbreak. The exponential growth rate was estimated to be 0.25 based on data collected from 15–23 January 2022.

Appendix Table 2. Estimated case-fatality-risk among the COVID-19 cases confirmed in the first 1–4 waves in comparison to cases with and without complete primary series who were identified in the early period of wave 5 (1 January – 15 February 2022) in Hong Kong.

	Waves 1 to 4			Wave 5 (Incomplete primary series) ‡			Wave 5 (Complete primary series)*		
Age group, y	Deaths	Recovered	CFR, % Mean (95% CI)	Deaths	Recovered	CFR, % Mean (95% CI)	Deaths	Recovered	CFR, % mean (95% CI)
<20	0	813	0.00 (0.00–0.45)	2	620	0.32 (0.04–1.16)	0	142	0.00 (0.00–2.56)
20–29	0	1,146	0.00 (0.00–0.32)	0	140	0.00 (0.00–2.60)	0	364	0.00 (0.00–1.01)
30–44	2	2,255	0.09 (0.01–0.32)	0	309	0.00 (0.00–1.19)	0	804	0.00 (0.00–0.46)
45–64	20	3,318	0.60 (0.37–0.92)	5	343	1.44 (0.47–3.32)	0	956	0.00 (0.00–0.39)
65–79	77	1,349	5.40 (4.28–6.70)	25	349	6.68 (4.37–9.71)	2	281	0.71 (0.09–2.53)
≥80	108	312	25.71 (21.60–30.18)	66	247	21.09 (16.70–26.03)	6	49	10.91 (4.11–22.25)

*Cases with complete primary series are individuals who have received at least 2 doses of COVID-19 vaccines before confirmation of infection, and cases with incomplete vaccinated refer to those without receiving any vaccine or with only one dose of vaccine.

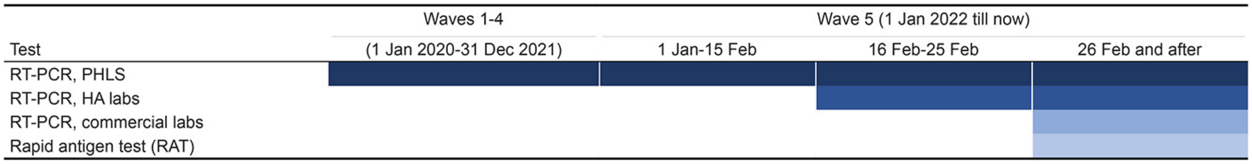

Appendix Figure 1. Laboratory confirmation of COVID-19 in Hong Kong over the epidemic waves since 2020. PHLS: The Public Health Laboratory Services Branch of Centre for Health Protection provides clinical diagnostic and public health laboratory services to the public and private health sectors for both patient care and public health functions. HA: The Hospital Authority is a statutory body managing all the government hospitals and institutes in Hong Kong.

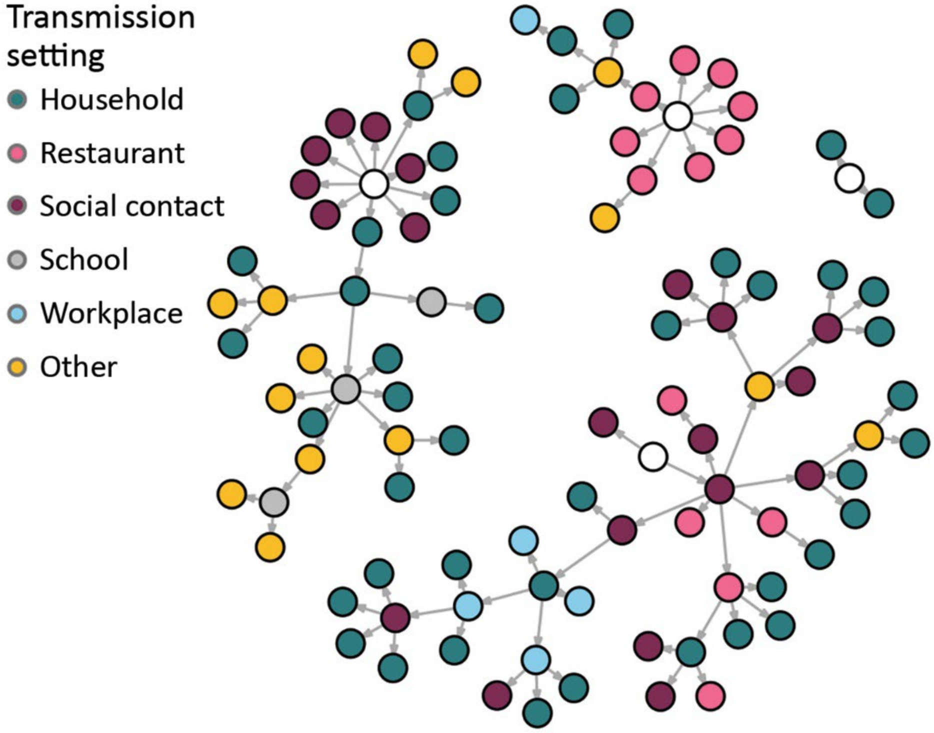

Appendix Figure 2. Transmission chains by transmission settings of SARS-CoV-2 Omicron infections associated with four imported cases in Hong Kong, 31 December 2021 to 22 January (n=98).

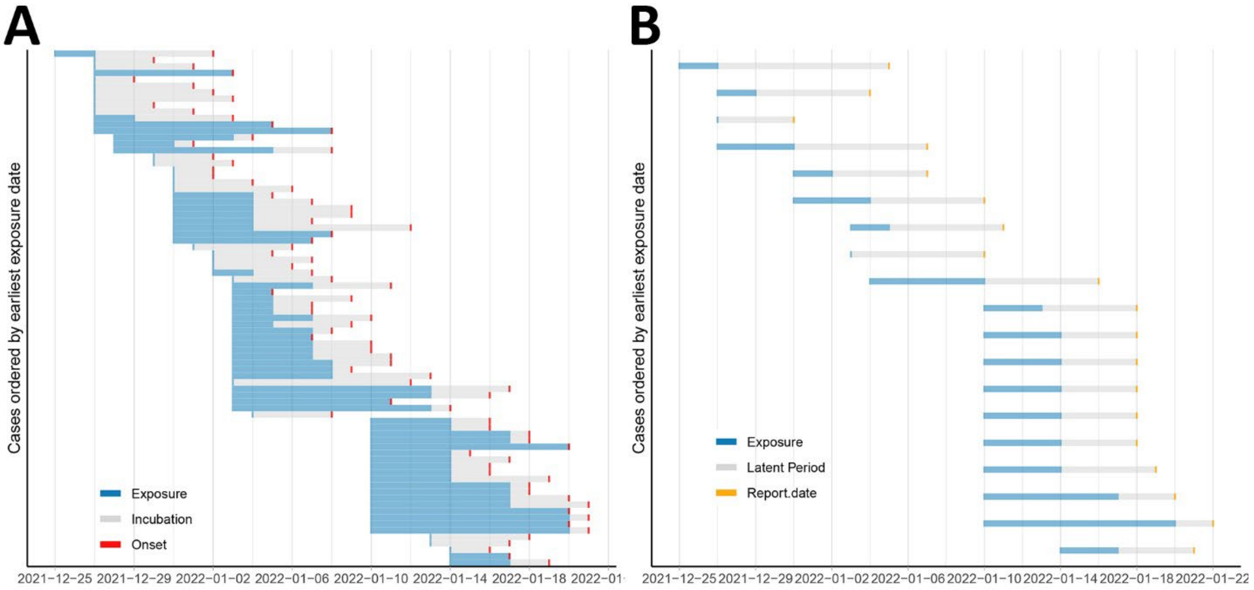

Appendix Figure 3. Cases (n=98) studied to estimate incubation period, serial interval and generation time of SARS-CoV-2 Omicron variant. Figure A refers to symptomatic cases and Figure B refers to asymptomatic cases. In each row, red shaded point indicates the dates of onset date, orange shaded point indicates confirmation date, blue shaded area indicates the period of exposure and that in grey indicates the incubation period.

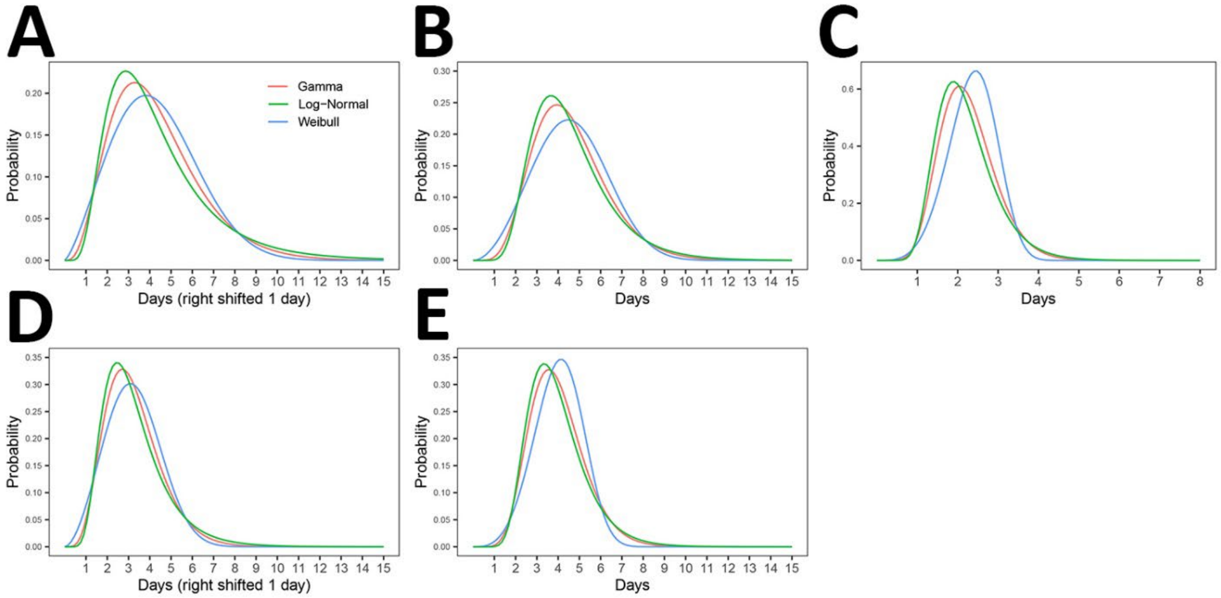

Appendix Figure 4. The estimated distributions of the incubation period, serial interval and generation time for infections occurred in infections with Omicron variant BA.1 and Omicron BA.2 in wave 5 in Hong Kong.
